# Giant cell tumor of bone following denosumab treatment: assessment of tumor response using various imaging modalities

**DOI:** 10.1186/s13244-020-00845-y

**Published:** 2020-02-27

**Authors:** Maram Alothman, Waleed Althobaity, Yasser Asiri, Saleh Alreshoodi, Khalid Alismail, Meshal Alshaalan

**Affiliations:** Medical Imaging Department, King Faisal Specialist Hospital and Research Centre, Riyadh, Kingdom of Saudi Arabia

**Keywords:** Giant cell tumor, Denosumab, Magnetic resonance imaging

## Abstract

**Background:**

Giant cell tumor (GCT) is a nonmalignant neoplasm composed of multinucleated giant and mononuclear stromal cells.

This study aimed to compare imaging findings of GCT pre- and post-denosumab treatment, including lesion size, percentage of signal intensity/density change, and time of initial objective tumor response. This will have a great impact on selection of most appropriate imaging technique to accurately measure therapy response and its related complications, which would influence the physicians to tailor the treatment regimen to suit each patient.

**Results:**

As per inverse Choi density/size (ICDS), 16 patients (84.2%) had an objective tumor response and 15 (78.9%) had an increase in density or decrease in signal intensity, and the mean of signal intensity decrease in the treated lesions was 32.4% (95% CI, 18–46.7). Only seven patients (36.8%) had tumors demonstrating ≥ 10% decrease in size, all of which showed a positive change in signal/density except for one. Moreover, 17 patients (89.4%) showed a clear demarcation/low signal intensity margin surrounding ≥ two third of the lesion periphery. The median time to first objective tumor response was approximately 23 weeks.

**Conclusion:**

Based on the ICDS criteria, most patients with giant cell tumor of bone show objective tumor response to denosumab. Modification of ICDS to include marginal sclerosis or clear demarcation of the lesions might be considered as a separate response criterion to accurately assess the treatment response in patients with GCT.

## Key points

- Inverse Choi density/size (ICDS) is a suitable and accurate method to assess tumor response to denosumab.
- MRI and plain radiographs detected tumor response, but many institutions prefer a combination of CT and plain radiograph.
- Most patients with giant cell tumor of bone show objective tumor response to denosumab.

## Background

Giant cell tumor (GCT) is a nonmalignant neoplasm composed of multinucleated giant and mononuclear stromal cells. The stromal cell population is a mesenchymal osteoblast precursor, which is the neoplastic component of GCT. GCT has an aggressive osteolytic nature related to activation of receptor activator of nuclear factor-kappa B ligand (RANKL) expressed by its giant cells [1].

GCT accounts for approximately 5% of all primary bone tumors and 20% of benign bone neoplasms in adults. Nearly half of most GCT lesions occur in the knee with a fewer than 3% of lesions seen in other sites such as the distal radius, proximal humerus, sacrum, and vertebral bodies [2].

After intralesional surgery combined with allograft or cement, the local recurrence rate of these lesions has been reduced to 12–14%. In nearly one tenth of patients, malignant transformation occurs at recurrence, and1–4% have pulmonary metastasis despite its benign histopathology [2].

Although surgery is considered as the standard treatment for GCT, patients with recurrent aggressive lesions or tumors at difficult anatomic locations are managed with alternative therapies [3].

Currently, denosumab is one of the treatment modality used in such challenging GCT cases. It is an FDA-approved monoclonal antibody that acts as RANKL inhibitor, which prevents bone destruction and induces sclerosis and remineralization [4].

RANKL plays a crucial role in GCT; it is expressed by the neoplastic stromal component and mediates recruitment of the monocytic precursors, which then develop osteoclast-like cells and erode bone [5].

In literature, several imaging studies such as plain radiographs, CT, MRI, and ^18^FDG-PET have been used to follow-up GCT treated with denosumab; however, data on the imaging criteria and the best modality to properly assess the treatment response are limited [6].

A few imaging assessment approaches to assess tumor response in bone neoplasms have been proposed in literature. Engellau et al. have suggested that the inverse Choi density/size (ICDS) and modified PET scan criteria are the most sensitive and accurate method to evaluate therapy response in GCT of bone (GCTB) [1].

This study aimed to compare imaging findings of GCT pre- and post-denosumab treatment, including lesion size, percentage of signal intensity/density change, and time of initial objective tumor response. This will have a great impact on selection of most appropriate imaging technique to accurately measure therapy response and its related complications, which would influence the physicians to tailor the treatment regimen to suit each patient.

## Method

This study reviewed the data of 20 patients with radiologically and pathologically proven GCTB treated with denosumab at KFSHRC, Riyadh, between January 2014 and May 2019. The exclusion criteria included absence of baseline or post-treatment imaging follow-up and the use of concurrent alternative treatment. One patient was excluded due to lack of baseline imaging. Patients were administered 120 mg denosumab subcutaneously every 4 weeks based on a standard treatment regimen. This study was approved by the Research Ethics Committee (REC) at KFSHRC.

The baseline clinical data, demographic profile, therapeutic regimen, and imaging findings on plain radiograph, CT scan, and MRI scan at baseline and 24-month follow-up were reviewed. MRI though was the mainstay modality used in this study. All cases were assessed in 1.5T MR scanners utilizing our center standard tumor protocol which included the following sequences: axial T1-weighted FSE, axial T2-weighted FSE with fat saturation, coronal STIR, axial T1-weighted GRE with fat saturation pre-contrast, and three planes post gadolinium contrast administration. Allowing for insignificant differences in TR, TE, and FOV, the remaining scanning parameters were consistent at baseline and follow-up.

The lesion size, textural/signal pattern, and time to first objective tumor response were evaluated using available modalities by two musculoskeletal radiologists blinded to the investigator assessment. The objective tumor response was assessed using the ICDS criteria. The lesion’s longest diameter and the drop of signal at follow-up were measured on T2 or STIR images as a percentage of the lesion overall surface area in most of the representative planes.

Objective tumor response was defined as either complete or partial response using ICDS as outlined in Table 1.

**Table 1:** Response criteria by inverse Choi density/size

Complete response	Disappearance of the disease
Partial response	A decrease in size (%Δ Choi SLD) ≥ 10% or an increase in CT density > 15% compared with baseline, no new lesions, and no obvious progression of non-measurable disease
Stable disease	Does not meet the criteria for CR, PR, or PD
Progressive disease	An increase in unidimensional tumor size (Choi SLD) ≥ 10% and does not meet the criteria for PR using CT density; any new lesions identified by CT/MRI; new intratumoral nodules or increase in the size of existing intratumoral nodules
Unevaluable	The CT/MRI exam is unavailable or deemed UE; if a target lesion is deemed UE by density and size measurement and the rules for PD do not apply, a response of CR, PR, or SD cannot be assigned for the time point and the response will be UE

*PR* partial response, *SLD* sum of longest diameter, *SD* stable disease, *PD* progressive disease, *UE* unevaluable

### Statistics

All the data were entered into a database and analyzed using the Statistical Package for the Social Sciences (SPSS 24.0), and descriptive statistics were presented. In order to determine the proportion of patients with objective tumor response and time to first objective tumor response on each imaging modality, a two-sided *p* value of less than 0.05 was considered to be statistically significant.

## Results

This study included 19 patients with an average age of 30.7 ± 10.2 years of whom nearly two third patients had primary GCT, one third had recurrence, and one patient had metastatic lesion. Most of the lesions were in the appendicular skeleton, and four target lesions were in the axial skeleton. Table 2 shows the baseline demographics and lesion characteristics.

**Table 2:** Demographic and clinical data of enrolled patients

Age		
Mean ± SD, range	30.7 ± 10.2, 48–16	
Sex		
Male	7	36.84%
Female	12	63.16%
Disease type		
Primary	15	78.95%
Recurrent	3	15.79%
Metastatic	1	5.26%
Location of primary lesion		
Pelvis/sacrum	2	10.53%
Lower extremity	8	42.11%
Upper extremity	7	36.84%
Spine	1	5.26%
Scapula	1	5.26%
Pre-denosumab treatment		
None	13	68.42%
Surgery	3	15.79%
Embolization	3	15.79%
Post-denosumab treatment		
None	7	36.84%
Surgery	11	57.89%
Embolization	1	5.26%

**Fig. 1:**
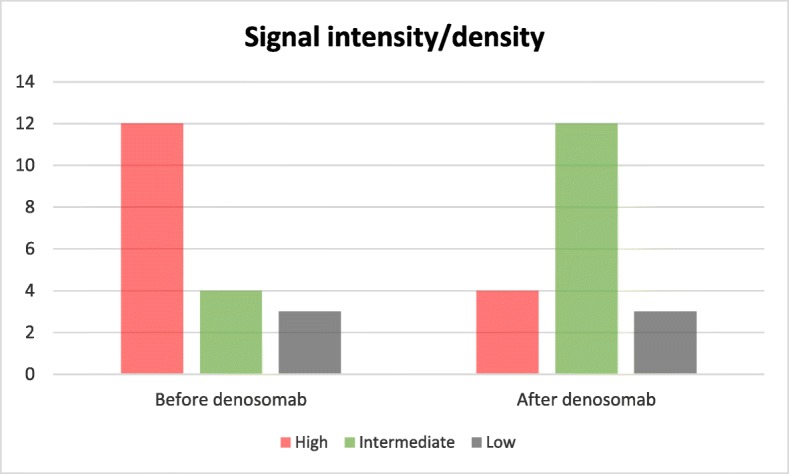
Pattern of signal alteration in treated lesions

**Fig. 2:**
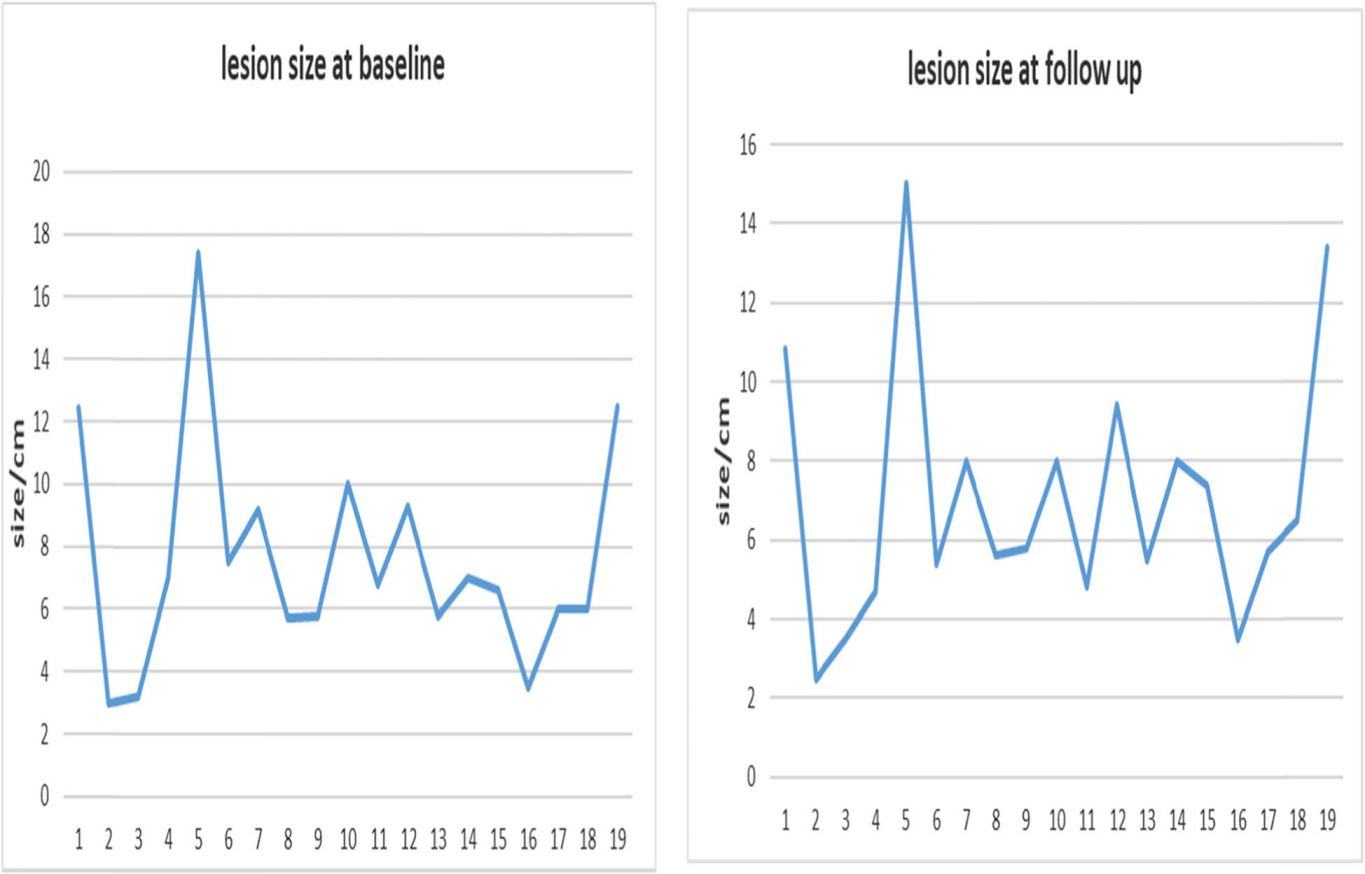
Lesions size pre and post therapy

As per ICDS, 16 patients (84.2%) had an objective tumor response and 15 (78.9%) had an increase in density or decrease in signal intensity, and the mean of signal intensity decrease in the treated lesions was 32.4% (95% CI, 18–46.7) as presented in Fig. 1. The longest diameter was used as a response measure parameter. Assessment of the interobserver variability revealed no systematic difference between observers. Only seven patients (36.8%) had tumors demonstrating ≥ 10% decrease in size, all of which showed a positive change in signal/density except for one (Fig. 2). Moreover, 17 patients (89.4%) showed a clear demarcation/low signal intensity margin surrounding ≥ two third of the lesion periphery. The median time to first objective tumor response was approximately 23 weeks. Almost half of the patients underwent surgical resection following treatment with no documented cases of recurrence. None of the patients developed pathological fracture or malignant transformation during or after the course of treatment. However, one case of osteonecrosis of the maxilla developed in a patient 3 years after the start of treatment which needed cessation of denosumab administration.

The three patients with no significant tumor response shared similar demographics with the rest of study subjects, for example, their average age was 24.4 ± 16.5 (*p* value 0.5) and their lesions were located at varying locations and nearly stable on subsequent follow-up imaging. Two of these patients underwent surgical resection.

**Fig. 3:**
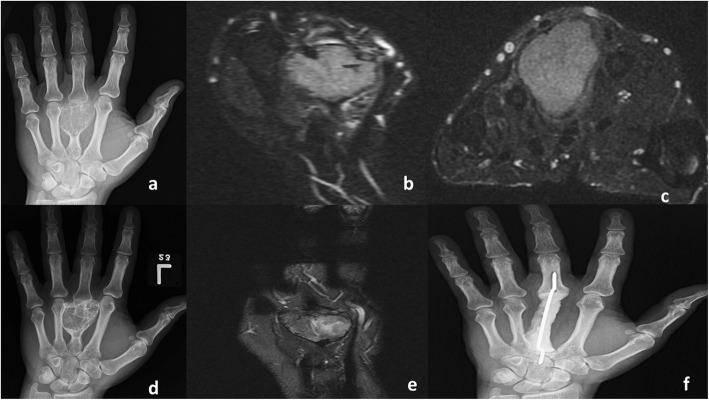
A 39-year-old male who was treated by denosumab for approximately 7 weeks then underwent en bloc surgical excision and non-biological reconstruction by cement/K-wire. Plain radiograph (**a**) and MRI (**b**) before treatment shows expansile metacarpal lytic lesion with corresponding high FS T2WI internal signal, and after treatment demonstrates marginal sclerosis and internal ossification on the X-ray (**d**) and newly developed areas of intermediate signal on follow-up MRI (**e**). Post-operative AP radiograph (**f**)

**Fig. 4:**
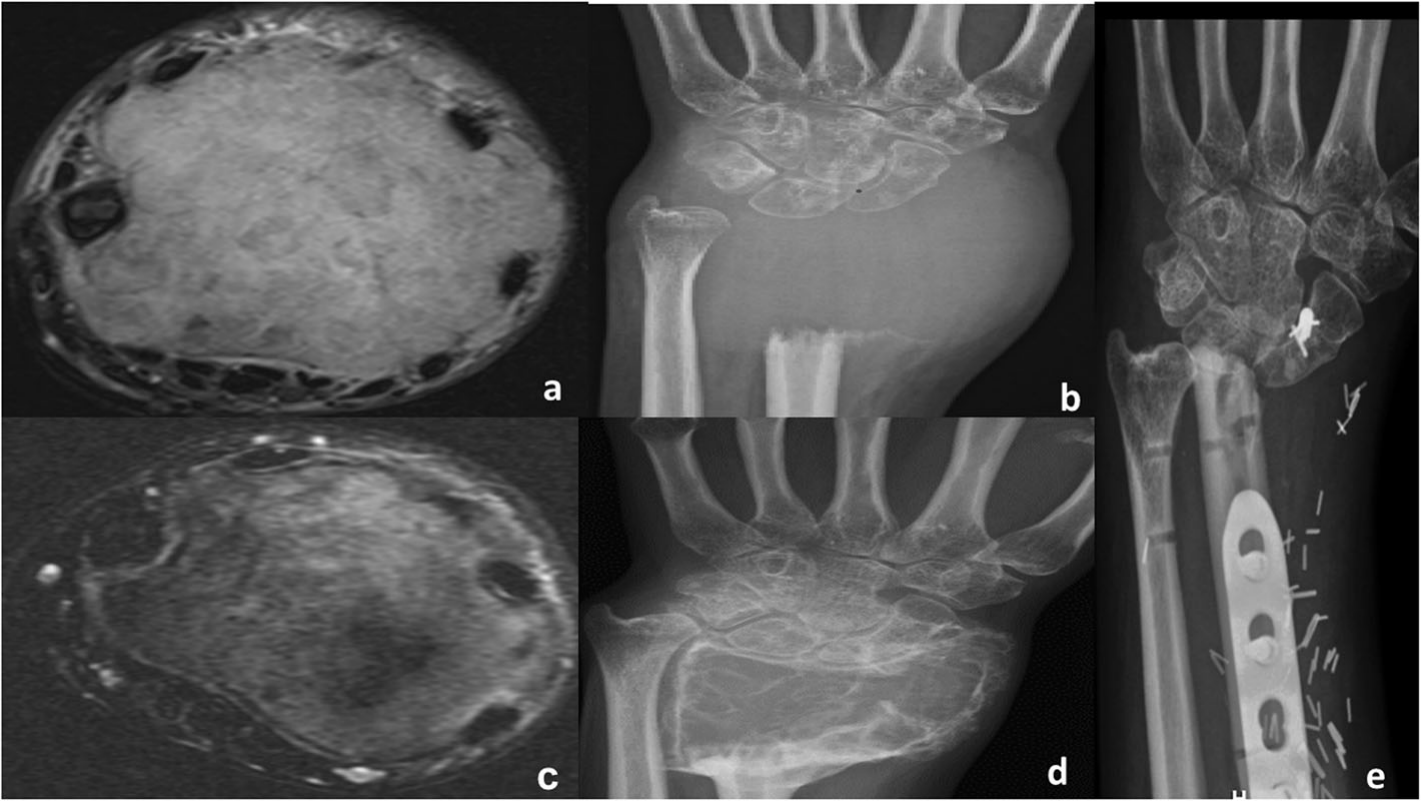
A 30-year-old female with giant cell tumor of distal radius treated with resection. MRI (**a**) and AP radiograph (**b**) before treatment show locally aggressive lytic lesion with ambiguous borders and after treatment (**c**, **d**) reveal internal mineralization of the lytic areas and clear sclerotic margin. Postoperative AP radiograph (**e**)

Two sets of MRI and plain radiograph images for patients with metacarpal and distal radius bone GCT before and after denosumab treatment are provided as an example for the expected findings in Figs. 3 and 4.

## Discussion

We detected a positive tumor response in the majority of patient with GCT following denosumab treatment using ICDS assessment criteria.

The pharmacodynamic response to denosumab is associated with change in lesion density rather than reduction in the size compared with other bone lesions. Consequently, RECIST alone is considered to be insensitive in evaluating treatment response of GCTB.

A few studies have compared various assessment methods and reported that ICDS is a suitable and more accurate alternative for demonstrating both pharmacodynamic and cytoreductive effects of denosumab in patients with GCTB [6, 7]. MRI and plain films were the consistent modalities for follow-up of these patients in our center. Unfortunately, no standard protocol was identified for imaging schedule or methodology, and CT was only used during image-guided biopsy of lesions. None of our patient was evaluated using PET scan. CT is the preferred follow-up imaging modality used by many institutions as it allows earlier detection of calcified peripheral margin and internal mineralization of GCT after denosumab therapy [4].

Modified PET scan criteria have proven to be another equally effective response assessment method to ICDS by similar studies done on a larger sample size, providing another promising alternative if PET scan is available in your centers [1].

The effectiveness of denosumab in reducing the stage of disease and sharpening the tumor margin prior to surgical resection has been established [2, 8]. In this study, denosumab was used to decrease the stage of the local disease prior to surgical resection in 57% of patients and control the progression of recurrent locally aggressive or unresectable lesions in the remaining patients. However, it does not prevent recurrence in patients who have been treated surgically previously [9].

Recurrence and metastasis to the lung and lymph nodes on baseline imaging were observed in three patients, and two patients achieved objective tumor response at the target lesions and metastatic lesions. Moreover, stable local disease and progression of the lung metastasis were observed in the third patient.

In patients with GCT, skeletal-related complications, such as pathological fracture and malignant transformation, with an incidence of approximately 30% and 2–5%, respectively, can occur especially after radiotherapy. Denosumab-related jaw osteonecrosis has a prevalence of 1.7% and can be detected through imaging and screening protocols that may be included for earlier detection. Other possible denosumab-related complications such as arthralgia, anemia, and hypocalcemia were not examined in our study [10–12].

A few case studies have reported development of osteosarcoma in patients with GCT following denosumab treatment [13–17]. None of these complications were encountered in our study as treatment-related issue. However, only one of the patients had denosumab-related osteonecrosis of the maxilla.

## Conclusion

Based on the ICDS criteria, most patients with GCTB show objective tumor response to denosumab. MRI and plain radiographs detected tumor response in our cases, but many institutions prefer a combination of CT and plain radiograph which seems to be a better alternative for a more accurate assessment considering the availability of HU as an objective measure. Modification of ICDS to include marginal sclerosis or clear demarcation of the lesions might be considered as a separate response criterion to accurately assess the treatment response in patients with GCTB.

## Data Availability

The datasets used and/or analyzed during the current study are available from the corresponding author on reasonable request.

## Abbreviations

AP: Anterior posterior
CT: Computed tomography
FDG-PET: 18-Fluoro-deoxyglucose positron emission tomography
FS T2W: Fat-saturated T2-weighted
FSE: Fast spin echo
GCT: Giant cell tumor
GRE: Gradient echo
ICDS: The inverse Choi density/size
KFSHRC: King Faisal Specialist Hospital and Research Center
MRI: Magnetic resonance imaging
RANK: Receptor activator of nuclear factor-kappa B
RECIST: Response Evaluation Criteria in Solid Tumors
STIR: Short tau inversion recovery
TE: Echo time
TR: Repetition time
